# Strong Genomic and Phenotypic Heterogeneity in the *Aeromonas sobria* Species Complex

**DOI:** 10.3389/fmicb.2017.02434

**Published:** 2017-12-08

**Authors:** Jeff Gauthier, Antony T. Vincent, Steve J. Charette, Nicolas Derome

**Affiliations:** ^1^Département de Biologie, Institut de Biologie Intégrative et des Systèmes, Université Laval, Quebec City, QC, Canada; ^2^Centre de Recherche de l’Institut Universitaire de Cardiologie et de Pneumologie de Québec, Quebec City, QC, Canada; ^3^Département de Biochimie, de Microbiologie et de Bio-informatique, Institut de Biologie Intégrative et des Systèmes, Université Laval, Quebec City, QC, Canada

**Keywords:** *Aeromonas sobria*, host–microbe interactions, bacterial genomics, microbial diversity, molecular systematics

## Abstract

*Aeromonas sobria* is a mesophilic motile aeromonad currently depicted as an opportunistic pathogen, despite increasing evidence of mutualistic interactions in salmonid fish. However, the determinants of its host-microbe associations, either mutualistic or pathogenic, remain less understood than for other aeromonad species. On one side, there is an over-representation of pathogenic interactions in the *A. sobria* literature, of which only three articles to date report mutualistic interactions; on the other side, genomic characterization of this species is still fairly incomplete as only two draft genomes were published prior to the present work. Consequently, no study specifically investigated the biodiversity of *A. sobria*. In fact, the investigation of *A. sobria* as a species complex may have been clouded by: (i) confusion with *A. veronii* biovar *sobria* because of their similar biochemical profiles, and (ii) the intrinsic low resolution of previous studies based on 16S rRNA gene sequences and multilocus sequence typing. So far, the only high-resolution, phylogenomic studies of the genus *Aeromonas* included one *A. sobria* strain (CECT 4245 / Popoff 208), making it impossible to robustly conclude on the phylogenetic intra-species diversity and the positioning among other *Aeromonas* species. To further understand the biodiversity and the spectrum of host-microbe interactions in *A. sobria* as well as its potential genomic diversity, we assessed the genomic and phenotypic heterogeneity among five *A. sobria* strains: two clinical isolates recovered from infected fish (JF2635 and CECT 4245), one from an infected amphibian (08005) and two recently isolated brook charr probionts (TM12 and TM18) which inhibit *in vitro* growth of *A. salmonicida* subsp. *salmonicida* (a salmonid fish pathogen). A phylogenomic assessment including 2,154 softcore genes corresponding to 946,687 variable sites from 33 *Aeromonas* genomes confirms the status of *A. sobria* as a distinct species divided in two subclades, with 100% bootstrap support. The phylogenomic split of *A. sobria* in two subclades is corroborated by a deep dichotomy between all five *A. sobria* strains in terms of inhibitory effect against *A. salmonicida* subsp. *salmonicida*, gene contents and codon usage. Finally, the antagonistic effect of *A. sobria* strains TM12 and TM18 suggests novel control methods against *A. salmonicida* subsp. *salmonicida*.

## Introduction

*Aeromonas* spp. is a genus of Gammaproteobacteria with substantial heterogeneity among species and subspecies in terms of environmental distribution, host range and growth conditions (Cahill, 1990; Janda and Abbott, 2010; Vincent et al., 2016). Aeromonads are ubiquitous in aquatic environments worldwide (Hazen et al., 1978; Chowdhury et al., 1990), either as (i) free-living organisms (Acinas et al., 1999), (ii) sessile life biofilms on biotic and abiotic surfaces (Talagrand-Reboul et al., 2017a) or (iii) as part of the natural microbiota of amphibians, mammals, reptiles and fish (Popoff and Véron, 1976; Cahill, 1990).

For aeromonads associated with fish hosts, there is a broad spectrum of symbiotic interactions, from mutualism (Gibson et al., 1998; Gunasekara et al., 2011) to pathogenicity (Austin and Austin, 2012a,b). The type of interaction can shift in a given host–microbe system, depending on overall host health and several environmental factors such as temperature, fish population density and water quality (Austin and Austin, 2012a,b). For instance, water temperatures above 17°C may trigger acute episodes of furunculosis (*A. salmonicida* subsp. *salmonicida*) in salmonids, with >90% mortality rates in less than a week post-infection (Scott, 1968), whereas for temperatures less than 12°C, fish may either be chronically infected (presence of skin nodules without mortalities) or become asymptomatic carriers (Schachte, 2002). Water temperature is indeed a critical factor, as climate change is predicted to elevate mean temperatures of some North American lakes and rivers to an optimum for *A. salmonicida* subsp. *salmonicida* growth (Tam, 2009; Tam et al., 2011).

Interactions of aeromonads in a given host-microbe system are further controlled by other microbial strains that colonize host body surfaces composing the so called microbiota. Among members of the microbiota, some strains are documented to exert antagonistic effects against pathogenic strains (Boutin et al., 2012, 2013; Goulden et al., 2012; Schubiger et al., 2015) including aeromonads (Zhang et al., 2016; Gao et al., 2017; Gauthier et al., 2017). Whether a host-associated aeromonad exerts mutualistic or pathogenic interactions is highly dependent on which host(s) it infects, but also its genetic repertoire, which varies greatly between strains/species (Ghatak et al., 2016).

In *A. salmonicida*, for example, there are five officially recognized subspecies (Dallaire-Dufresne et al., 2014). Three of them (*achromogenes*, *masoucida* and *smithia*) infect a broad range of hosts including cod (*Gadus morhua*), black rockfish (*Sebastes schlegeli*) and turbot (*Scophtalmus maximus*) (Cornick et al., 1984; Larsen and Pedersen, 1996; Han et al., 2011). Subspecies *pectinolytica* is without any report of pathogenicity, and subspecies *salmonicida* almost exclusively infects salmonid fish (Austin and Austin, 2012a). The broad host range of *A. salmonicida*, as well as the presence of mesophilic strains in this mainly psychrophilic species (Vincent et al., 2016), are evidence of a great genomic heterogeneity and complexity. Indeed, the *A. salmonicida* pangenome (i.e., total non-redundant genes among 26 strains) is made of 8,164 genes, of which 59.2% are accessory genes (Vincent and Charette, 2017). The *A. salmonicida* pangenome is “open,” suggesting a high prevalence of genetic material exchanges across other bacteria sharing the same environment (Rouli et al., 2015).

Similarly, motile aeromonads *A. hydrophila, A. veronii* and *A. caviae* also exhibit open pangenomes with a high species-wise proportion of accessory genes (61.7%, 53.4%, and 50.9% respectively), with strong variation in terms of antimicrobial resistance and virulence genes (Ghatak et al., 2016). *A. media*, noted for its remarkable genomic and phenotypic heterogeneities, shows the highest known species-wise proportion of accessory genes for an *Aeromonas* species (68.4%) (Talagrand-Reboul et al., 2017b).

Genome “openness,” i.e., strong heterogeneity and high exchangeability of genes, seems to be a defining trait of this genus. This strong genomic heterogeneity, revealed by next-generation sequencing, has led to major reclassifications in the *Aeromonas* taxonomy (Beaz-Hidalgo et al., 2013, 2015). Indeed, of all 30 *Aeromonas* species with valid taxonomic status, half were described since the 2005 edition of the Bergey’s Manual of Systematic Bacteriology (Boone et al., 2005). Novel species continue to be described (Figueras et al., 2017).

However, there are other relevant *Aeromonas* species complexes whose diversity and complexity has not been as thoroughly characterized. To this respect, one example of interest is *Aeromonas sobria* (*sensu* Popoff and Véron, 1976), a mesophilic motile aeromonad currently depicted as an opportunistic pathogen of freshwater fish, amphibians and reptiles (Wahli et al., 2005; Janda and Abbott, 2010; Austin and Austin, 2012b; Yang Q.-H. et al., 2017). In spite of increasing evidence of mutualistic interactions mediated by *A. sobria* strains (Brunt and Austin, 2005; Brunt et al., 2007; Pieters et al., 2008), the determinants of its host-microbe associations remain less understood than for other aeromonad species such as *A. salmonicida* subsp. *salmonicida* (Garduño and Kay, 1992; Garduño et al., 1993; Vanden Bergh and Frey, 2013). Indeed, the current literature on *A. sobria* is not only scarce with respect to other aeromonads, but is also strongly biased by an over-representation of pathogenic interactions.

From 1981 to date, PubMed referenced 142 articles dedicated to *A. sobria* while ISI Web of Science referenced 140 *A. sobria* articles from 1978 to date. Articles specifically discussing *A. veronii* biovar *sobria* are not included in this estimate. This constitutes about 10 times less literature than for *A. hydrophila*, a well-documented fish and human opportunistic pathogen (Cipriano et al., 1984; Janda and Abbott, 2010) and about five times less literature than for *A. salmonicida* subsp. *salmonicida*, a major pathogen of salmonids (Austin and Austin, 2012a; Dallaire-Dufresne et al., 2014). There are only three reports on mutualistic interactions by *A. sobria* (Brunt and Austin, 2005; Brunt et al., 2007; Pieters et al., 2008), all of which dealt with the same *A. sobria* strain (GC2).

This rough estimate of 140 *A. sobria* articles may be lower: prior to the description of *A. veronii* (Hickman-Brenner et al., 1987), several ornithine-decarboxylase negative *A. veronii* strains (now referred to as *A. veronii* biovar *sobria*) were incorrectly labeled as *A. sobria*. As a consequence, epidemiology prior to 1987 may be unreliable to this regard. Ironically, the majority of those articles, referenced in either PubMed or Web of Science, are outbreak reports of immunocompromised human patients. Unlike *A. veronii*, few *A. sobria* (*sensu stricto*) strains have been isolated from sources other than fish and aquatic environments (Janda and Abbott, 2010).

Next-generation sequencing data on *A. sobria* is also scarce. Prior to this publication, only two genome assemblies (both drafts) were available on GenBank, compared to the 61 entries for *A. hydrophila* and 36 for *A. salmonicida*. The genome of the type strain CECT 4245 was sequenced through a large-scale study on the genus *Aeromonas* (Colston et al., 2014) while the one of 08005 was published as a Genome Announcement (Yang Q.-H. et al., 2017). Consequently, no study specifically investigated the genomic features and diversity of *A. sobria*.

To increase our knowledge about the spectrum of host–microbe interactions in *A. sobria* as well as its biodiversity, we report the comparative phenotypic and genomic analysis of five host-associated *A. sobria* strains including two mutualistic strains with strong *in vitro* antagonistic effect against *A. salmonicida* subsp. *salmonicida*. Genome sequencing and comparative analyses of these strains revealed unexpected heterogeneity between all five *A. sobria* strains in terms of phylogeny, codon usage and gene contents, which closely correlates with their phenotype regarding their inhibitory effect against *A. salmonicida* subsp. *salmonicida*.

## Materials and Methods

### Bacterial Isolates and Growth Conditions

*Aeromonas sobria* strains TM12 and TM18 were both isolated in 2015 from the intestinal contents of an adult brook charr (*Salvelinus fontinalis*) from Lake Prime-Huron, Quebec, Canada. Strain JF2635 was isolated in 2001 from a European perch (*Perca fluviatilis*) in Switzerland (Wahli et al., 2005). Strain CECT 4245 (formerly known as Popoff 208) was isolated from an infected fish specimen (Popoff and Véron, 1976). All *A. sobria* isolates were grown on lysogeny broth (LB) agar or Tryptic Soy Agar plates (TSA, BD Diagnostics) plates at 18°C or 30°C, except recently sequenced strain 08005 (Yang Q.-H. et al., 2017) which was only included in the comparative genomic analyses. Indeed, this novel genome sequence (08005) was published after *in vitro* assays were completed. Given the scarcity of *A. sobria* genome sequences, we chose to include this strain in comparative genomic analyses, despite the absence of *in vitro* results, in order to maximize taxon sampling.

### Phenotypic Characterization

#### Interspecific Antagonism Assays

Bacterial lawns of ten *A. salmonicida* subsp. *salmonicida* strains (Supplementary Table 1) were prepared by streaking a sterile swab dipped in liquid culture (OD_600_ = 0.7) on TSA plates. Wells were punched in the agar using sterile pipette tips with a diameter of 3.5 mm. For each *A. sobria* isolate, 10 μL of liquid culture (OD_600_ = 0.7) were dispensed in an assigned well. Plates were incubated at 18°C for 96 h. Inhibition surfaces around the wells were measured on 23.6 pixel/mm scans with software ImageJ version 1.48k (Schneider et al., 2012), with the well area subtracted from the whole inhibition area.

#### Antimicrobial Activity of Extracellular Products (ECP)

For each *A. sobria* isolate, ECPs were recovered by centrifuging overnight liquid cultures incubated at 18°C for 24 h in LB broth, all adjusted to OD_600_ = 0.7. Culture supernatants (CS), obtained by centrifugation at 4,000 × *g* for 20 min at 4°C, were then filtered with a 0.2 μm Filtropur S syringe disk filter (Sarstedt), arrayed on a Bioscreen C microplate (Growth Curves AB Ltd, Helsinki, Finland), and supplemented with an equal volume of *A. salmonicida* subsp. *salmonicida* 01-B526 liquid culture in LB broth adjusted at OD_600_ ∼ 0.05. *A. salmonicida* subsp. *salmonicida* 01-B526 CS and fresh LB medium were used as neutral and negative controls for *A. sobria* CSs, respectively. Mixtures were incubated in a Bioscreen C plate reader at 18°C for 48 h, with OD_600_ measured at each hour. Statistical significance of growth differences between conditions was assessed with a one-way ANOVA for repeated measures over time (*df_time_* = 47, *df_conditions_* = 4, α = 0.05). Data used for testing were balanced (i.e., equal number of observations per condition), and respected the assumptions of normality (Shapiro–Wilk test: W = 0.98407, *p* = 0.9899) and homoscedasticity (Bartlett’s test: *K*^2^ = 2.2485, *df* = 4, *p* = 0.6902). *Post hoc* comparisons of means were performed using Tukey’s HSD test only if a statistically significant difference was detected by ANOVA.

#### Biofilm Formation

The ability of *A. sobria* to produce biofilms in liquid broth was verified with the microtiter dish assay described by O’Toole (2011) with minor modifications. Briefly, overnight LB broth cultures adjusted at OD_600_ = 0.9 were diluted 1:100 in either LB-Miller or Tryptic Soy Broth (TSB, BD Diagnostics); 100 μL of each diluted culture were arrayed in triplicates on disposable PVC U-bottomed plates (VWR International). Plates were incubated at 30°C without shaking for 6 h. After incubation, OD_600_ was measured to estimate bacterial abundance in each culture. Biofilms were stained by adding 25 μL of 1% aqueous Crystal Violet solution in each well, and were let standing for 15 min at room temperature (RT). Wells were rinsed abundantly with distilled water. Wells were then washed twice with 200 μL 95% ethanol, which was kept and arrayed on a clean microtiter plate. Biofilms were quantified by reading the OD_600_ in each ethanol/Crystal Violet mixture. Statistical significance of growth differences between conditions was assessed with a two-way ANOVA (Factors: Growth media and *A. sobria* strain, *df_media_* = 1, *df_strain_* = 4, α = 0.05). Data used for testing were balanced (i.e., had an equal number of observations per condition), and were log_10_-transformed to improve normality (Shapiro–Wilk test: *W* = 0.90401, *p* = 0.01054) and homoscedasticity (Levene’s test: *df* = 9, *F* = 1.0206, *p* = 0.4571). No other data transformation improved normality and homoscedasticity as efficiently as the log_10_ transformation. *Post hoc* comparisons of means were performed using Tukey’s HSD test only if a statistically significant difference was detected by ANOVA.

#### Growth Kinetics

For each *A. sobria* isolate, 400 μL of overnight culture in LB adjusted at OD_600_ ∼ 0.05 were arrayed on a sterile transparent covered plate (CORNING). The plate was incubated at 30°C in an Infinite 200 PRO microplate incubator/reader equipped with a 595 nm absorbance filter (TECAN, Morrisville, NC, United States). The plate was shaken (200 RPM) for 48 h; OD_595_ was measured at each incubation cycle of 15 min.

### Comparative Genomics Analyses

#### DNA Extraction and Genome Sequencing

The total genomic DNA of *A. sobria* strains JF2635, TM12, and TM18 was extracted using a DNeasy Blood and Tissue Kit (Qiagen, Canada). The sequencing libraries were prepared using a KAPA Hyper Prep Kit and were sequenced by next-generation sequencing (NGS) on a MiSeq instrument (Illumina technology) by the Plateforme d’Analyse Génomique of the Institut de Biologie Intégrative et des Systèmes (IBIS, Université Laval). The resulting sequencing reads were *de novo* assembled into contiguous sequences using the A5-miseq pipeline version 20160825 (Tritt et al., 2012). Contigs were ordered using mauveAligner (Rissman et al., 2009) with the complete genome of *Aeromonas veronii* B565 (CP002607.1) as a reference. The complete draft genomes were annotated with RAST (Aziz et al., 2008) and the NCBI Prokaryotic Genome Annotation Pipeline (PGAP) and deposited in the public database GenBank (TM12: NQML00000000, TM18: NQMM00000000, and JF2635: LJZX00000000). The whole genome sequence of *A. sobria* CECT 4245 (GenBank: CDBW00000000.1) and 08005 (GenBank: NZ_MKFU00000000) were already available prior to this study.

#### Plasmid Assembly and Annotation

The high-copy plasmid sequences were recovered by downsampling the sequencing reads using seqtk^1^ before re-performing *de novo* assemblies with the A5-miseq pipeline. The plasmid sequences were annotated with the RAST web server (Aziz et al., 2008) and were manually curated. The presence or absence of a type II toxin-antitoxin locus was assessed for each sequence using TAfinder (Shao et al., 2011). Plasmid sequences were deposited on GenBank (MF770238 and MF770239 for strain JF2635; MF770240, MF770241, and MF770242 for strain TM18).

#### Molecular Systematics

In addition to three *A. sobria* genome sequences produced by the present study, 30 genome sequences from representative strains of all *Aeromonas* species available in GenBank were downloaded, thus making a dataset of 33 genomes (Supplementary Table 2). To avoid annotation bias, all the sequences were locally annotated with Prokka version 1.12-beta (Seemann, 2014). Homology links between the coding sequences were detected with GET_HOMOLOGUES version 20170105 (Contreras-Moreira and Vinuesa, 2013) using two algorithms, COG (Kristensen et al., 2010) and OMCL (Li et al., 2003). Homologous sequences detected with both algorithms were kept for the subsequent analyzes. The 2,154 nucleotidic sequences corresponding to orthologous genes of the softcore (genes present in at least 95% of the genomes) and without paralogous ambiguity were codon aligned by muscle version 3.7 (Edgar, 2004) through TranslatorX (Abascal et al., 2010). Monomorphic sites were removed for each alignment with BMGE version 1.2 (Criscuolo and Gribaldo, 2010). All the sequences were concatenated and partitioned into a supermatrix by AMAS (Borowiec, 2016). The best-fit model was found for each partition using IQ-TREE version 1.5.3 (Nguyen et al., 2015). Finally, a maximum-likelihood tree was inferred also using IQ-TREE and the branch supports obtained with 10,000 ultrafast bootstraps (Minh et al., 2013). The average nucleotide identity (ANI) was computed for the 33 taxa using pyani (Pritchard et al., 2016) and NUCmer version 3.1 (Kurtz et al., 2004).

#### Other Analyses

The pangenome of all five *A. sobria* strains studied here was inferred using GET_HOMOLOGUES (Contreras-Moreira and Vinuesa, 2013), allowing to sort the genes in four categories based on orthologous gene cluster frequency distribution: core genes (present in all genomes), softcore (present in 95% of all genomes), cloud (present in 1 or 2 genomes only) and shell (all remaining genes). Relative Synonymous Codon Usage (RSCU) was computed using DAMBE6 (Xia, 2017). The principal components analysis (PCA) used to differentiate the isolates in two groups based on the RSCU values was performed by the R package ade4 (Dray and Dufour, 2007). Antibiotic resistance genes were found using the resistance gene identifier (RGI) from the CARD database (McArthur et al., 2013). Genes implicated in a secretion system were found by TXSScan (Abby et al., 2016). Prophages were detected with PHASTER (Arndt et al., 2016) using pseudo-finished genome assemblies prepared with CONTIGuator v2.7.1 (Galardini et al., 2011) using the *A. veronii* B565 complete genome (CP002607.1) as a reference chromosome for contig alignments.

## Results and Discussion

### Strong Phenotypic Heterogeneity

#### Interspecific Antagonism

Two of the *A. sobria* strains (TM12 and TM18) analyzed in this study are gut symbionts recovered from healthy brook charr (*Salvelinus fontinalis*) (**Table 1**). Both were isolated in a research project aiming to study the interactions between resident brook charr bacteria and *A. salmonicida* subsp. *salmonicida*. Both strains had strong, yet qualitatively different *in vitro* inhibitory effects against fish pathogen *A. salmonicida* subsp. *salmonicida* (**Table 2**). This finding was interesting because two *A. sobria* strains were recovered from healthy specimens, yet also had an inhibitory effect against *A. salmonicida* subsp. *salmonicida* which is also part of the resident brook charr microbiota (Dallaire-Dufresne et al., 2014). This prompted the assessment of this antagonistic effect in other *A. sobria* strains (JF2635, CECT 4245), which are clinical isolates recovered from infected fish (**Table 1**). No data regarding their inhibitory effect against *A. salmonicida* subsp. *salmonicida* was available prior to this study.

**Table 1:** *Aeromonas sobria* strains used in this study.

Strain	Source organism	Host disease status	Year of isolation	Reference
TM12	Brook charr (*Salvelinus fontinalis*)	Healthy	2015	This study
TM18	Brook charr (*Salvelinus fontinalis*)	Healthy	2015	This study
JF2635	European perch (*Perca fluviatilis)*	Moribund	2004	a
CECT 4245^T^	Fish (unknown)	Unknown	ca. 1967–74	b
08005 ^∗^	American bullfrog (*Rana castebeiana)*	Moribund	2016	c


*a: Wahli et al. (2005). b: Popoff and Véron (1976). c: Yang Q.-H. et al. (2017). T, type strain (CECT, 1991); duplicated from Popoff 208 (1976). ^∗^Strain present only in genomic analyses.*

**Table 2:** Diffusible inhibitory effect of *A. sobria* strains on TSA bacterial lawns of *A. salmonicida* subsp. *salmonicida*, after 96 h at 18°C.

*A. salmonicida* subsp. *salmonicida*		*A. sobria*			
	
Origin	Strain	TM12	TM18	JF2635	CECT 4245^T^
Province of Quebec, Canada	01-B522	+	+++	–	–
	01-B526	+	++	–	–
	09-0167	++	+++	–	–
	M15879-11	+	+	–	–
	m23067-09	+	+++	–	–
New Brunswick, Canada	04-05MF26	++	+++	–	–
	09-144K3	++	+++	–	–
Norway	HER1085	++	+++	–	–
Switzerland	JF2267	+	+	–	–
France	A449^T^	+	+++	–	–


*+ 50–100 mm^*2*^; ++ 100–200 mm^*2*^; +++ more than 200 mm^*2*^; – no visible inhibition plaque; T, type strain.*

While *A. sobria* isolates TM12 and TM18 had an antagonistic effect against 10 strains of *A. salmonicida* subsp. *salmonicida* from various hosts and geographical origins (**Table 2**), CECT 4245 and JF2635 did not show any disruptive effect on the growth of any *A. salmonicida* subsp. *salmonicida* strain, even after 96 h. Radial inhibition on *A. salmonicida* subsp. *salmonicida* bacterial lawns by TM12 and TM18 suggests involvement of diffusible inhibitory compounds, even though major differences were observed between these two strains. Strain TM12 produced inhibition halos overlapping the diffusion area of a blue pigment while TM18 did not exhibit any visible pigmentation, but a significantly stronger antagonistic effect (Supplementary Figure 1). Production of inhibition zones on *A. salmonicida* subsp. *salmonicida* lawns indicates that *A. sobria* TM12 and TM18 (but neither JF2635 nor CECT 4245) can produce diffusible antimicrobial compounds that inhibit *A. salmonicida* subsp. *salmonicida.* Interestingly, both strains that were isolated as causative infectious agents (JF2635 and CECT 4245) had no inhibitory effect against *A. salmonicida* subsp. *salmonicida*, whereas TM12 and TM18 (recovered from asymptomatic fish) had a strong antimicrobial effect on *A. salmonicida* subsp. *salmonicida.*

The production of antimicrobial compounds targeting *A. salmonicida* subsp. *salmonicida* has also been assessed by exposing *A. salmonicida* subsp. *salmonicida* 01-B526 to *A. sobria* extracellular products (ECPs) in culture supernatants (Supplementary Figure 2). Interestingly, results do not follow the trend observed in the agar assays described above. In fact, no significant change of *A. salmonicida* growth was detected after 48 h growth [*F*_(4,10)_ = 0.482, *p* = 0.749].

It is possible that the inhibitory compounds produced in agar are not produced when grown in liquid broth. Indeed, in solid medium assays, *A. sobria* strains were in conditions of high cell density without direct contact with *A. salmonicida* subsp. *salmonicida* cells, i.e., conditions resembling a bacterial biofilm (McBain, 2009). On the opposite, in liquid broth assays, *A. sobria* cells were in conditions more akin to planktonic life before recovery of their ECPs (i.e., in liquid broth with continuous shaking). Therefore, the growth characteristics of *A. sobria* isolates in liquid broth were investigated.

#### Growth Kinetics

**FIGURE 1:**
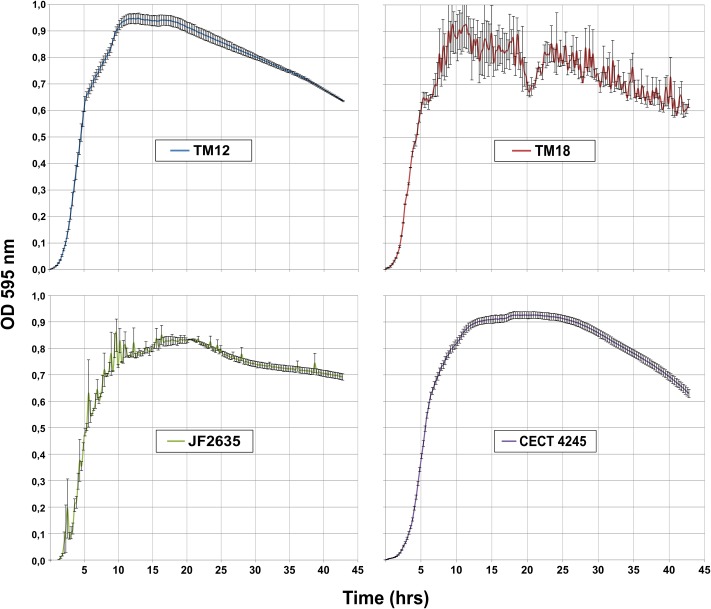
Growth kinetics of *A. sobria* strains in LB broth at 30°C (in triplicates).

All *A. sobria* strains undergo a similar growth pattern in LB broth (**Figure 1**). Stationary phase is reached after 10 h (OD_600_ ∼ [0.8; 0.9]), followed by gradual decline. However, one striking difference is the high levels of background noise in the TM18 growth curve throughout the stationary and decline phases. This suggests that either cell aggregation occurs in liquid cultures, or that TM18 has the ability to form significant levels of biofilms in liquid broth. The latter possibility was subsequently assessed.

#### Biofilm Formation

**FIGURE 2:**
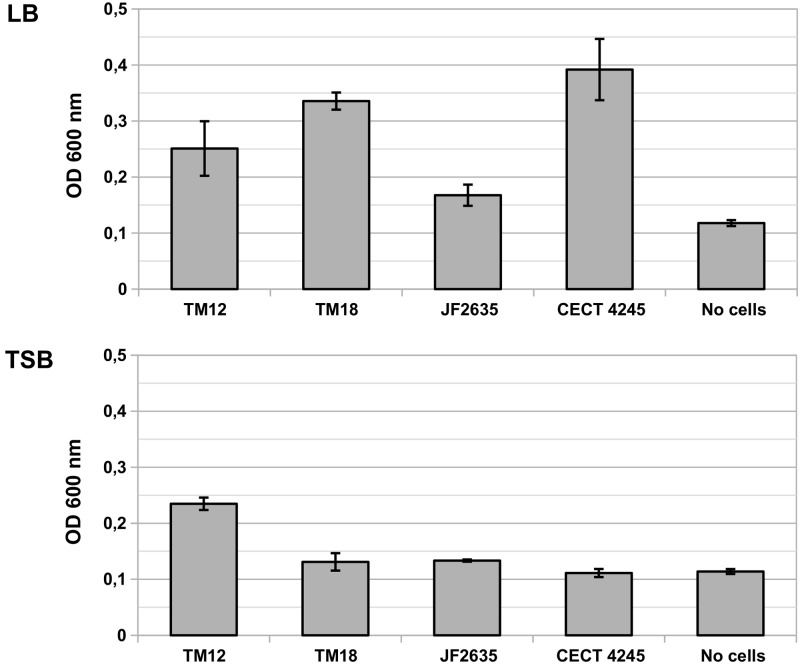
Biofilm production in liquid cultures of *A. sobria* over 6 h static incubation at 30°C in LB and TSB broth. Bars indicate the OD_600_ of crystal violet that adsorbed in biofilms. Vertical segments indicate the standard error of the mean. This experiment was performed in triplicates.

The levels of biofilm production by *A. sobria* are significantly different in LB than in TSB media [*F*_(1,20)_ = 62.80, *p* = 1.35 × 10^-7^], and vary significantly between strains [*F*_(4,20)_ = 17.87, *p* = 2.20 × 10^-6^) (**Figure 2**). There is a strong interaction between the growth medium and the ability of *A. sobria* strains to produce biofilms [*F*_(4,20)_ = 16.30, *p* = 4.39 × 10^-6^).

*Post hoc* multiple comparisons revealed that *A. sobria* strains TM12, TM18 and CECT 4245 produced significant levels of biofilm compared to the no-cell control (Tukey’s HSD test, *p* ≤ 0.021), but with marginal between-strain differences (Tukey’s HSD test, *p ≥* 0.084). Biofilm-producing strains do better in LB than in TSB (Tukey’s HSD test, *p* = 10^-7^). Strain JF2635 produced no detectable amount of biofilm over the no-cell control (Tukey’s HSD test, *p* = 0.65).

RAST genome annotations of *A. sobria* strains revealed that strains TM18 and CECT 4245 lack the *pga* operon required for the biosynthesis of biofilm adhesin poly-β-1,6-N-acetyl-D-glucosamine (**Table 3**). This finding suggests that (i) TM18 and CECT 4245 could be more vulnerable to compounds inhibiting surface attachment (i.e., surfactants), and (ii) certain nutrients present in TSB broth but not in LB broth (i.e., enzymatic soymeal digest or glucose), could act as inhibitors of biofilm formation. Indeed, mono- and diglycerides are known surfactants (Prajapati et al., 2012) and inhibitors of biofilm formation in *Aeromonas* (Ham and Kim, 2016) that can be biologically synthesized in high abundance from enzymatic soymeal digests, due to its high phospholipid content (Küllenberg et al., 2012).

**Table 3:** Differential presence of genes involved in biofilm synthesis and glycerophospholipid catabolism, with special relevance to biofilm formation in *A. sobria*.

Category	RAST annotation	Major product/function	*A. sobria*			
			
			TM12	TM18	JF2635	CECT 4245
Biofilm adhesin biosynthesis	*pgaA* (Biofilm PGA outer membrane secretin)	Outer membrane PGA	P		P	
	*pgaC* [Biofilm PGA synthesis *N*-glycosyl transferase (EC 2.4.-.-)]	GlcNAc export to periplasm	P		P	
	*pgaB* (Biofilm PGA synthesis deacetylase (EC 3.-))	Promotes PGA export through the PgaA porin	P		P	
Glycero- phospholipid Catabolism	Phospholipase C 4 precursor (EC 3.1.4.3)	DAG^∗^ + phospholipid head group		P		P
	Putative phospholipase A1-like (EC 3.1.1.32)	Lysophospholipid^∗^ + free fatty acid	P	P	P	P
	Lysophospholipase L2 (EC 3.1.1.5)	Free fatty acid	P	P		P

Biofilm formation in LB			+	++	-	++
Biofilm formation in TSB			+	-	-	-


*PGA, Poly-beta-1,6-*N*-acetyl-*D*-glucosamine. DAG, diacylglycerol. GlcNAc, *N*-acetyl glucosamine. EC, Enzyme Commission number for enzymes. P, present. +, weak biofilm formation. ++, strong biofilm formation. -, no biofilm production. ^∗^known surfactants.*

Interestingly, *A. sobria* strains possess several phospholipase genes that could produce surfactant metabolites (**Table 3**). Strains TM12, TM18 and CECT 4245 are likely able to produce (i) diacylglycerol (DAG) *via* phosphatidylcholine-specific phospholipase C, and (ii) free fatty acids *via* lysophospholipase L2. The latter are known to play a role as signal molecules involved in either biofilm formation or dispersion (Marques et al., 2015), and could lead to biofilm inhibition. Strain JF2635 (which produces no biofilm in either LB or TSB) possesses a phospholipase A1 gene but lacks lysophospholipase. In a liquid broth rich in glycerophospholipids such as TSB, this may result in a buildup of extracellular lysophospholipids which have strong detergent properties (Huang et al., 1998).

### Heterogeneity in the *A. sobria* Species Pangenome

There is significant quantitative and qualitative heterogeneity among the four *A. sobria* strains of this study in terms of basic phenotypic traits such as (i) antagonism against another *Aeromonas* species; (ii) growth kinetics and iii) production of biofilms in different growth conditions. Knowing that those four strains were isolated from fairly different backgrounds (**Table 1**), this heterogeneity may be underlain by strong genomic divergence resulting from adaptation to different niches. It was therefore tempting to verify if the phylogenomic clustering among the *A. sobria* strains studied here also reflects this heterogeneity.

#### Core and Accessory Genomes

The pangenome of *A. sobria* strains studied here exhibits a species-wise proportion of accessory genes of 2,084/5,586 = 37.3% (**Table 4**). This is lower than reported values for other *Aeromonas* species complexes, where accessory genes represent 50–70% of the pangenome (see Introduction). This low proportion can be explained by the scarcity of sequence data for *A. sobria* (five genomes including those introduced in this publication). Indeed, a development plot of the core genome (i.e., a fit of core genome size *vs.* number of subsampled genomes) reveals that an asymptote has not yet been reached (i.e., the number of core genes will decrease by adding more genomes; Supplementary Figure 3A). Conversely, a development plot shows that the pangenome is “open,” i.e., it would increase if more *A. sobria* genomes were included in the study (Supplementary Figure 3B) (Guimarães et al., 2015; Rouli et al., 2015). This suggests much of the phylogenomic diversity of *A. sobria* remains to be assessed, which will be solved by the addition of more whole genome data.

**Table 4:** Core and accessory gene counts across *A. sobria* strains of this study.

*A. sobria*	Cloud	Shell	Soft core	Core	Total
08005	461	169	3,502	3,339	4,132
CECT 4245	455	170	3,502	3,339	4,127
JF2635	667	161	3,425	3,339	4,330
TM12	412	93	3,455	3,339	3,960
TM18	505	142	3,471	3,339	4,118
Total ^∗^	2,084		3,502	3,339	5,586


*Cloud: present in 1 or 2 genomes only. Core: present in all genomes. Softcore: present in 95% of all genomes. Shell: all remaining genes. ^∗^Column totals are not necessarily sums of counts for each strain, since certain elements are shared between one or more strains.*

#### Molecular Phylogeny

**FIGURE 3:**
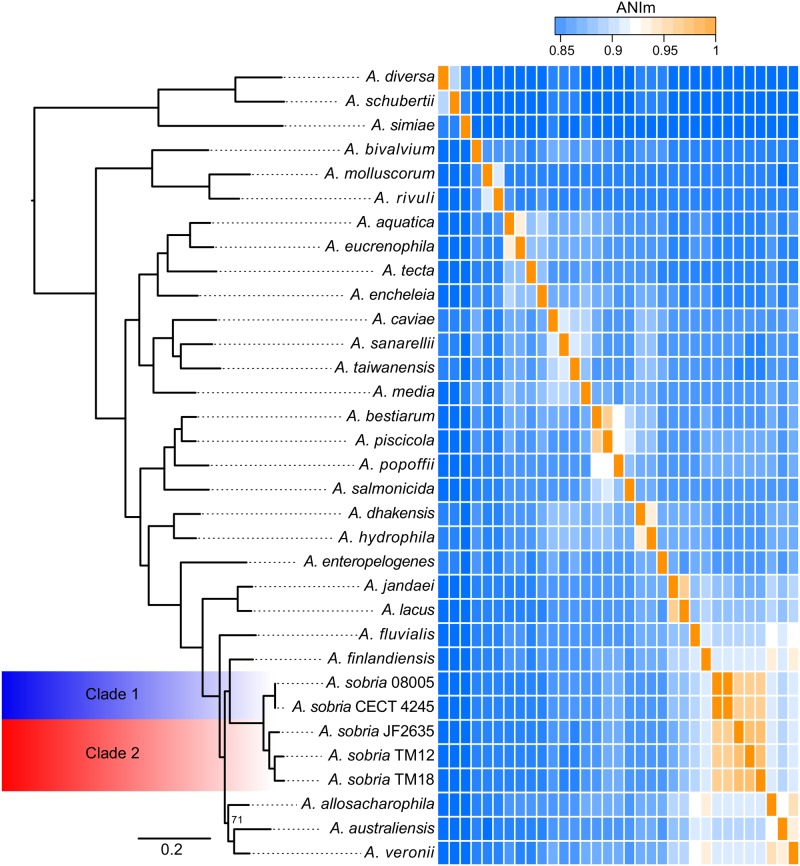
Phylogenomic tree of the *Aeromonas* softcore genome (2,154 genes present in at least 95% of 33 *Aeromonas* genomes), coupled to an ANIm analysis of the *Aeromonas* genus with an emphasis on the *sobria* species. All nodes are supported by bootstrap values of 100, excepted the one of *allosaccharophila*, which is 71. The ANIm heatmap is a square matrix; rows and columns are ordered identically.

In addition to the *A. sobria* isolates, a set of representative strains of each *Aeromonas* species with whole genome sequences available in GenBank (Supplementary Table 2) has been added to get a more accurate phylogenetic resolution of the *A. sobria* isolates. This includes the genome sequences of a fifth *A. sobria* strain, 08005. This additional strain was recovered from an infected amphibian. As expected, the five *A. sobria* isolates formed a monophyletic group (**Figure 3**).

As mentioned earlier, there is apparent confusion between *A. sobria sensu stricto* (Popoff and Véron, 1976) and *A. veronii* biovar *sobria* in the scientific literature, because of the similarity in their phenotypic profiles (Janda and Abbott, 2010; Austin and Austin, 2012b). However, the softcore genome phylogeny supports that *A. veronii* and *A. sobria* are distinct clades (**Figure 3**), as previously demonstrated by other clustering methods (Martino et al., 2011). To our knowledge, our phylogenetic assessment, which is based on 2,154 softcore gene sequences including 946,687 variable sites of 33 *Aeromonas* genomes, is the most robust and accurate phylogenetic positioning of *A. sobria* to date.

Interestingly, the *sobria* clade shares a near common ancestor with the *A. finlandiensis* species. Multilocus sequence analysis trees (7 and 15 housekeeping genes) from the paper having reported this species placed it near the species *A. allosaccharophila* and *A. veronii*, while *A. sobria* was more basal (Beaz-Hidalgo et al., 2015). A recent study, based on two concatenated gene sequences reported *A. sobria* forming a clade along with *A. allosaccharophila* while *A. veronii* was predicted to share a recent common ancestor with the one of *A. finlandiensis* (Sanglas et al., 2017). In addition to the present study, two papers describing phylogenies of the *Aeromonas* genus based on core and softcore genomes have been published (Colston et al., 2014; Vincent et al., 2016). Unfortunately, these two publications did not include *A. finlandiensis* because those two studies were initiated prior to its description (Beaz-Hidalgo et al., 2015). We believe that more complete genomes of strains from the *A. finlandiensis* species is required to have a clearer taxonomic positioning relative to *A. sobria*.

#### Average Nucleotide Identity

The average nucleotide identity (ANI) is known to be a gold standard to determine the relatedness of bacterial species, where a value of ∼95–96% correlates with the ∼70–75% DNA:DNA hybridization threshold used as a gold standard to define prokaryotic species (Konstantinidis and Tiedje, 2005; Goris et al., 2007; Colston et al., 2014; Federhen et al., 2016). The ANI values confirmed that the five *A. sobria* isolates are members of the same species (**Figure 3**). This analysis, in addition to the short phylogenetic branch lengths, showed that the strains 08005 and CECT 4245 (hereby referred to as “Clade 1”) are evolutionarily close (Shared ANI: 99.9%). It is worth noting that strains JF2635, TM12 and TM18 (hereby referred to as “Clade 2”), which all grouped together in the softcore phylogeny, exhibited substantial nucleotide diversity (Shared ANI: 96.4 ± 0.4%). The split corresponding to both clades is strongly supported with a bootstrap score of 100.

#### Codon Usage

**FIGURE 4:**
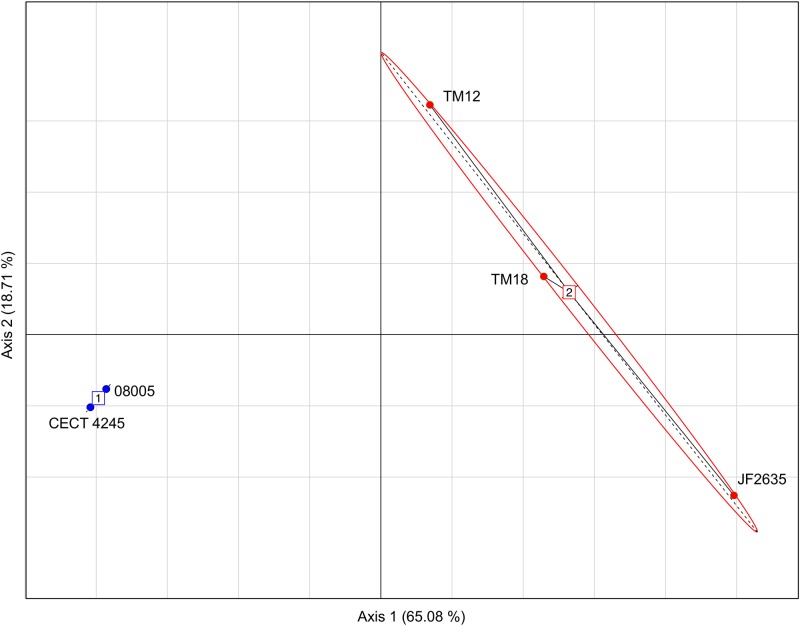
Principal components analysis (PCA) based on the RSCU values showing a separation between isolates from clade 1 (blue) and those from clade 2 (red).

The genomic dissimilarity underlying the split into two clades was striking as it resulted mostly from the number of tRNA genes encoded by these genomes (**Table 5**). Clade 1 isolates harbored 20–22% less tRNA genes than clade 2. Given this extensive dichotomy in tRNA genes, it was reasonable to hypothesize that some codons could be preferred, depending on the clade. The relative synonymous codon usage (RSCU) was found for each set of genes and the result was analyzed by a principal component analysis (PCA) in which the isolates were distributed as expected as in the phylogenetic tree (i.e., in two distinct groups), suggesting that a codon bias exists depending on the clade (**Figure 4**). Furthermore, the PCA also confirmed the more important heterogeneity within clade 2, previously evidenced in other analyses.

**Table 5:** Genome sequences of *Aeromonas sobria* strains used in the present study.

Strain	Contigs	N50 (pb)	Coverage (x)	GC (%)	CDS	tRNA	GenBank	Ref.
TM12	98	136 123	112.26	57.81	4036	115	NQML00000000	This study
TM18	101	127 327	109.57	57.72	4256	116	NQMM00000000	This study
JF2635	121	128 869	75.04	57.84	4426	115	LJZX00000000	This study
CECT 4245	48	171 779	34	57.62	4223	91	NZ_CDBW00000000	a
08005	52	186 724	109.0	57.58	4219	90	NZ_MKFU00000000	b


*a: Colston et al. (2014). b: Yang L. et al. (2017).*

#### Antibiotic Resistance Genes

**FIGURE 5:**
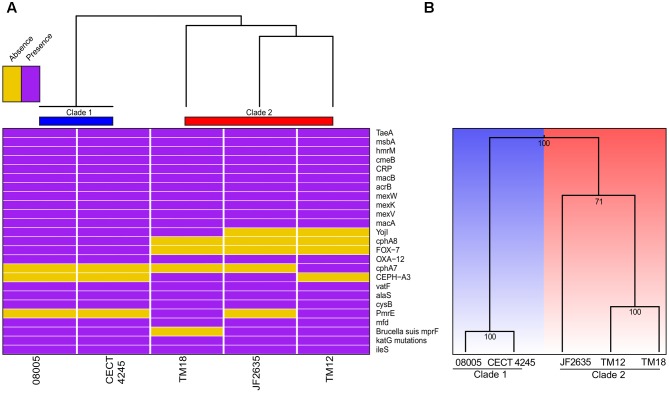
**(A)** Heatmap based on the presence or absence of the antibiotic resistance genes. **(B)** Clustering based on the presence or absence of genes implicated in secretion systems.

Bacterial genomes harbor various key genes to enhance their fitness, including drug resistance and virulence factors. In *A. sobria*, little is known about the pool of coding genes used for antibiotic resistance mechanisms and to colonize new environments. Thorough genomic sequence investigation allowed to identify several genes conferring drug resistance (**Figure 5A**), many of which are coding either for efflux pumps or beta-lactam resistance proteins. This was not unsuspected knowing that aquatic environments are favorable for the spread of antibiotic resistance genes (Baquero et al., 2008), and that aeromonads are documented to be effective vectors for such genes (Henriques et al., 2006; Vincent et al., 2014; Piotrowska and Popowska, 2015; Trudel et al., 2016). Interestingly, there was a congruence regarding the phylogenetic signal between antibiotic resistance genes and the overall genomic sequence (i.e., supporting the same clade 1 and clade 2 dichotomy). The sole incongruence concerns the cluster 2 root: JF2635 roots the whole genome based phylogeny while TM18 roots the antibiotic resistance genes-based clustering (**Figure 5A**). Even if it is perilous to draw conclusions about evolutionary history of resistance genes in *A. sobria* given the small number of markers comparatively to the molecular phylogeny, the fact that both topologies are similar lets us believe that resistance genes are not mobile and are stable.

#### Virulence Factors

Secretion systems are well characterized as sophisticated protein machineries widely distributed among bacteria and are, among other things, major determinants in virulence (Costa et al., 2015; Abby et al., 2016; Green and Mecsas, 2016). As for antibiotic resistance genes, it was consequently relevant to verify the presence or absence of genes implicated in these systems (Supplementary Table 4). Here, the clustering analysis of virulence genes was even more congruent with the whole genome phylogeny than what was observed for resistance genes (**Figure 5B**). One of the most salient features was the absence of mandatory genes involved in the formation of a T6SSi, a secretion system exporting effectors to both bacterial and eukaryotic cells (Ho et al., 2014), for clade 2 isolates. A functional T6SS was previously reported in *A. hydrophila* (Suarez et al., 2008). Then, the five *A. sobria* genomes were predicted to harbor all mandatory genes for functional T1SS, T2SS, type IV pilus (T4P) and flagellum (Supplementary Table 5). Also the genomes of 08005, CECT 4245 and JF2635 were predicted to bear the single mandatory gene (coding for a protein having both the translocator and passenger domains) to have a functional T5aSS (Leo et al., 2012).

#### Plasmids

Of all five *A. sobria* strains included in this study, only two (TM18 and JF2635 from clade 2) harbored small high-copy-number plasmids (Supplementary Figure 4). TM18 has three plasmids ranging from 4,393 to 5,190 bp, whilst JF2635 harbors two plasmids (3,818 and 5,381 bp). Plasmids pJF2635-1 and pTM18-3 are ColE1-like replicons, as evidenced by the presence of genes encoding regulatory RNAs I and II involved in ColE1-type replication (Tomizawa, 1984). The other plasmids (pJF2635-2, pTM18-1 and pTM18-2) are ColE2-type replicons which have no RNA II gene but a RNA I gene complementary to the *repA* mRNA (Sugiyama and Itoh, 1993).

Aside from RNA I and RNA II genes, most genes found in the plasmid repertoire of *A. sobria* TM18 and JF2635 encode either hypothetical proteins or proteins involved in plasmid mobility and maintenance (Supplementary Figure 4; blue arrows). Two notable exceptions are:

(i) a putative *vapD* gene in pTM18-2, which may encode a virulence-associated protein with ssRNA endonuclease activity (Katz et al., 1992; Kwon et al., 2012). No other *vap* gene homologs were found in neither the plasmids nor the draft genome sequences.
(ii) a pseudogene in pTM18-1. A BLASTx search against the non-redundant protein database (NCBI) returned a hypothetical protein from an *Aeromonas* sp. as best hit. No putative conserved domain was detected.

#### Prophages

A total of nine predicted phage elements were found across the five *A. sobria* isolates (Supplementary Table 3). Seven of those prophages were found in JF2635, of which only two were presumably intact: a Phi018p-like element (Beilstein and Dreiseikelmann, 2008) and a SJ46-like element (Yang L. et al., 2017). Only one intact prophage, a Fels-2-like element is found in both CECT 4245 and 08005 strains (clade 1), whilst only one prophage, a 9.5 kb Phi018p-like element (presumably incomplete) was found in both TM12 and TM18 strains (clade 2). There is a clear dichotomy in terms of prophage contents between strains from clade 1 (CECT 4245 and 08005) and strains from clade 2 (TM12, TM18 and JF2635). Even among clade 2 strains, there is a split, with JF2635 having substantially more phage elements (Supplementary Figure 5). The presence of many degenerated elements in JF2635 suggests that this strain acquired prophages early in the evolutionary history of clade 2. The low number of phage elements in other *A. sobria* strains, as opposed to JF2635, requires further investigation.

## Conclusion

*Aeromonas sobria* is a mesophilic motile aeromonad whose host–microbe associations, either mutualistic or pathogenic, are less understood than for other aeromonad species. We assessed the genomic and phenotypic heterogeneity among five *A. sobria* strains: two brook charr probionts (TM12 and TM18) which inhibit *in vitro* growth of *A. salmonicida* subsp. *salmonicida*, and three clinical isolates recovered from infected fish (JF2635 and CECT 4245) and an infected amphibian (08005). Comparative analysis supports a split of the *A. sobria* species complex in two clades.

• Clade 1 strains (08005 and CECT 4245) harbor no plasmids but a single intact Fels-2-like prophage. They also possess an identical antibiotic resistance gene profile. They have no inhibitory effect against *A. salmonicida* subsp. *salmonicida*.
• Clade 2 strains (TM12, TM18 and JF2635) possess 20–22% more tRNA genes than clade 1 strains, leading to major differences in relative synonymous codon usage. They harbor no Fels-2-like prophage, but minimally an incomplete Phi018p-like prophage. There are notable differences between clade 2 strains, however. Unlike TM12 and TM18, strain JF2635 has two intact and five incomplete prophages. Only TM18 and JF2635 harbor plasmids. Their antibioresistance gene and secretion gene profiles are more heterogeneous than within clade 1. Only clade 2 strains inhibit growth of *A. salmonicida* subsp. *salmonicida* (TM12 and TM18), with the exception of JF2635.

These findings illustrate how adaptation to a broad range of hosts and life strategies has shaped the evolution of the *A. sobria* species complex into two clades harboring significant within-clade and between-clade diversity. *A. sobria* has been treated as a monotypic bacterial species since its inception (Popoff and Véron, 1976; Martinez-Murcia et al., 1992; Abbott et al., 2003; Martino et al., 2011). However, the clear genomic and phenotypic division between clade 1 and clade 2 indicates that the *A. sobria* species complex may be composed of at least two candidates to subspecies status. Of course, the taxonomic assessment of *A. sobria* below the species level will be more accurate when more genome sequences will be available.

Finally, the antagonistic effect of clade 2 strains TM12 and TM18 against *A. salmonicida* subsp. *salmonicida* indicates that these strains (or their products) could lead to novel control and prevention methods to mitigate this opportunistic pathogen of salmonid fish. This effect suggests a role of certain host-associated *A. sobria* strains in controlling the abundance of other opportunistic pathogens in their host microbiota (including other aeromonads) and deserves further investigation.

## Author Contributions

JG, AV, SC, and ND designed the experiments. JG and AV performed *in vitro* and *in silico* experiments. JG, AV, SC, and ND contributed to the manuscript.

## Conflict of Interest Statement

The authors declare that the research was conducted in the absence of any commercial or financial relationships that could be construed as a potential conflict of interest.

## Footnotes

1 https://github.com/lh3/seqtk
