# Supplementary material for: Strong Genomic and Phenotypic Heterogeneity in the Aeromonas sobria Species Complex
Source: Front Microbiol. 2017 Dec 8;8:2434. doi: 10.3389/fmicb.2017.02434 (PMC5727048; doi:10.3389/fmicb.2017.02434)
Supplement: Supplementary file 1 [file Data_Sheet_1.docx]

Supplementary Material

Strong Genomic and Phenotypic Heterogeneity
in the *Aeromonas sobria* Species Complex

Jeff Gauthier*, Antony T. Vincent, Steve J. Charette and Nicolas Derome

*** Correspondence:** Jeff Gauthier, [jeff.gauthier.1@ulaval.ca](mailto:jeff.gauthier.1@ulaval.ca)

Contents

[Supplementary Tables 2](#_Toc494101472)

[Supplementary Table 1. *Aeromonas salmonicida* subsp. *salmonicida* strains used in this study. 2](#_Toc494101473)

[Supplementary Table 2. *Aeromonas* genomes used in the core genome phylogeny. 3](#_Toc494101475)

[Supplementary Table 3. Characteristics of predicted prophages in *A. sobria* genomes. 4](#_Toc494101476)

[Supplementary Table 4. Antibiotic resistance genes detected in *A. sobria* strains of this study with the Resistance Gene Identifier (CARD). 5](#_Toc494101477)

[Supplementary Table 5. Protein secretion genes detected in *A. sobria* strains of this study with TXSScan. 7](#_Toc494101478)

[Supplementary Figures 10](#_Toc494101479)

[Supplementary Figure 1. Diffusible inhibitory effect of *A. sobria* strains TM12 and TM18 against *A. salmonicida* subsp. *salmonicida* 09-0167 lawns on TSA plates. 10](#_Toc494101480)

[Supplementary Figure 2. Growth of *A. salmonicida* 01-B526 in [3:1] mixtures of LB broth and *A. sobria* culture supernatants (CS). 11](#_Toc494101481)

[Supplementary Figure 3. Plasmidome of the *A. sobria* isolates. 15](#_Toc494101482)

[Supplementary Figure 4. Heatmap of predicted phage elements in *A. sobria* genomes. 16](#_Toc494101483)

# Supplementary Tables

| **Strain** | **Isolate** | |
| --- | --- | --- |
|  | **Host Species** | **Origin** |
|  |  |  |
| 01-B522 | *Salvelinus fontinalis* | Canada |
| HER1085 | unknown | Norway |
| 01-B526 | *Salvelinus fontinalis* | Canada |
| 09-0167 | *Salmo salar* | Canada |
| *2004-05 MF26* | unknown | Canada (NB) |
| *2009-144 K3* | *Salvelinus fontinalis* | Canada (NB) |
| A449 | *Salmo trutta* | France |
| JF2267 | *Salvelinus alpinus* | Switzerland |
| M15879-11 | *Salvelinus fontinalis* | Canada |
| m23067-09 | *Salvelinus fontinalis* | Canada |

## Supplementary Table 1. *Aeromonas salmonicida* subsp. *salmonicida* strains used in this study.

NB, New-Brunswick. All other Canadian Isolates are from the Province of Quebec.

## Supplementary Table 2. *Aeromonas* genomes used in the core genome phylogeny.

| **Species** | **Strain** | **GenBank Accession** |
| --- | --- | --- |
| *A. allosaccharophila* | CECT 4199 | NZ_CDBR00000000 |
| *A. aquatica* | AE235 | NZ_JRGL01000000 |
| *A. australiensis* | CECT 8023 | NZ_CDDH00000000 |
| *A. bestiarum* | CECT 4227 | NZ_CDDA00000000 |
| *A. bivalvium* | CECT 7113 | NZ_CDBT00000000 |
| *A. caviae* | YL12 | NZ_JOVP00000000 |
| *A. dhakensis* | AAK1 | NZ_BAFL00000000 |
| *A. diversa* | 2478-85 | NZ_APVG00000000 |
| *A. encheleia* | CECT 4342 | NZ_CDDI00000000 |
| *A. enteropelogenes* | CECT 4255T | NZ_CDDE00000000 |
| *A. eucrenophila* | CECT 4224 | NZ_CDDF00000000 |
| *A. finlandiensis* | 4287D | NZ_JRGK00000000 |
| *A. fluvialis* | LMG 24681 | NZ_CDBO00000000 |
| *A. hydrophila* | ATCC 7966 | NC_008570 |
| *A. jandaei* | CECT 4228 | NZ_CDBV00000000 |
| *A. lacus* | AE122 | NZ_JRGM00000000 |
| *A. media* | WS | NZ_CP007567 |
| *A. molluscorum* | 848T | NZ_AQGQ00000000 |
| *A. piscicola* | LMG 24783 | NZ_CDBL00000000 |
| *A. popoffii* | CIP 105493 | NZ_CDBI00000000 |
| *A. rivuli* | DSM 22539 | NZ_CDBJ00000000 |
| *A. salmonicida* | A449 | NC_009348 |
| *A. sanarellii* | LMG 24682 | NZ_CDBN00000000 |
| *A. schubertii* | WL1483 | NZ_CP013067 |
| *A. simiae* | CIP 107798 | NZ_CDBY00000000 |
| *A. sobria* | 08005 | NZ_MKFU01000000 |
| *A. sobria* | CECT 4245 | CDBW00000000 |
| *A. sobria* | JF2635 | LJZX00000000 |
| *A. sobria* | TM12 | NQML00000000 |
| *A. sobria* | TM18 | NQMM00000000 |
| *A. taiwanensis* | LMG 24683 | NZ_BAWK00000000 |
| *A. tecta* | CECT 7082 | NZ_CDCA00000000 |
| *A. veronii* | B565 | NC_015424 |

## Supplementary Table 3. Characteristics of predicted prophages in *A. sobria* genomes.

|  | | | | | **Presence / Absence** | | | | |
| --- | --- | --- | --- | --- | --- | --- | --- | --- | --- |
| **PHAST Most Common Phage ID** | **Phage Family *** | **Length (kb)** | **# ORFs** | **GC %** | **TM12** | **TM18** | **JF2635** | **CECT 4245** | **08005** |
| Fels_2 | Myo. | 42 | 59 | 55.8 | - | - | - | + | + |
| RS138 | Siph. | 21.6 | 9 | 57.5 | - | - | ± | - | - |
| 118970_sal3 | Myo. | 20.9 | 24 | 51.5 | - | - | ± | - | - |
| phiO18P (I) | Myo. | 55.9 | 51 | 59.2 | - | - | + | - | - |
| phiO18P (II) | Myo. | 18.1 | 21 | 48.7 | - | - | ± | - | - |
| phiO18P (III) | Myo. | 9.5 | 11 | 59.1 | ± | ± | - | - | - |
| RSA1 | Myo. | 13.6 | 21 | 57.1 | - | - | ~ | - | - |
| S_SKS1 | Siph. | 6.3 | 9 | 38.3 | - | - | ± | - | - |
| SJ46 | Myo. | 42.1 | 65 | 54.9 | - | - | + | - | - |

+, present and presumably complete. ±, present but presumably incomplete. ~, present but completeness unknown. -, absent. * Phage families were found from the NCBI taxonomies of the most common phage IDs. Myo., Myoviridae. Siph., Siphoviridae. ORF, open reading frame.

## Supplementary Table 4. Antibiotic resistance genes detected in *A. sobria* strains of this study with the Resistance Gene Identifier (CARD).

| **CARD annotation** | **TM12** | **TM18** | **JF2635** | **CECT 4245** | **08005** | **Category** |
| --- | --- | --- | --- | --- | --- | --- |
| *taeA* | 1 | 1 | 1 | 1 | 1 | efflux pump conferring antibiotic resistance |
| *msbA* | 1 | 1 | 1 | 1 | 1 | efflux pump conferring antibiotic resistance |
| *hmrM* | 1 | 1 | 1 | 1 | 1 | efflux pump conferring antibiotic resistance |
| *cmeB* | 1 | 1 | 1 | 1 | 1 | efflux pump conferring antibiotic resistance |
| *CRP* | 1 | 1 | 1 | 1 | 1 | efflux pump conferring antibiotic resistance; gene modulating antibiotic efflux |
| *macB* | 1 | 1 | 1 | 1 | 1 | efflux pump conferring antibiotic resistance |
| *acrB* | 1 | 1 | 1 | 1 | 1 | efflux pump conferring antibiotic resistance |
| *mexW* | 1 | 1 | 1 | 1 | 1 | efflux pump conferring antibiotic resistance |
| *mexK* | 1 | 1 | 1 | 1 | 1 | efflux pump conferring antibiotic resistance |
| *mexV* | 1 | 1 | 1 | 1 | 1 | efflux pump conferring antibiotic resistance |
| *macA* | 1 | 1 | 1 | 1 | 1 | efflux pump conferring antibiotic resistance |
| *yojI* | 0 | 1 | 0 | 1 | 1 | efflux pump conferring antibiotic resistance |
| *cphA8* | 0 | 0 | 0 | 1 | 1 | antibiotic inactivation enzyme; beta-lactam resistance protein |
| *FOX-7* | 0 | 0 | 0 | 1 | 1 | antibiotic inactivation enzyme; beta-lactam resistance protein |
| *OXA-12* | 1 | 1 | 1 | 1 | 1 | antibiotic inactivation enzyme; beta-lactam resistance protein |
| *cphA7* | 1 | 0 | 0 | 0 | 0 | antibiotic inactivation enzyme; beta-lactam resistance protein |
| *CEPH*-A3 | 0 | 1 | 1 | 0 | 0 | antibiotic inactivation enzyme; beta-lactam resistance protein |
| *vatF* | 1 | 1 | 1 | 1 | 1 | antibiotic inactivation enzyme; streptogramin resistance protein |
| aminocoumarin resistant *alaS* | 1 | 1 | 1 | 1 | 1 | aminocoumarin resistant alaS |
| aminocoumarin resistant *cysB* | 1 | 1 | 1 | 1 | 1 | aminocoumarin resistance protein |
| *pmrE* | 1 | 1 | 0 | 0 | 0 | polymyxin resistance protein; gene altering cell wall charge |
| *mfd* | 1 | 1 | 1 | 1 | 1 | antibiotic target protection protein; fluoroquinolone resistance protein |
| *Brucella suis mprF* | 1 | 0 | 1 | 1 | 1 | antibiotic target modifying enzyme; peptide antibiotic resistance protein |
| Mycobacterium tuberculosis *katG* mutations conferring resistance to isoniazid | 1 | 1 | 1 | 1 | 1 | antibiotic resistant gene variant or mutant; isoniazid resistance protein |
| Bifidobacteria intrinsic *ileS* conferring resistance to mupirocin | 1 | 1 | 1 | 1 | 1 | Bifidobacteria intrinsic ileS conferring resistance to mupirocin |

CARD, Comprehensive Antimicrobial Resistance Database. 1, present. 0, absent.

## Supplementary Table 5. Protein secretion genes detected in *A. sobria* strains of this study with TXSScan.

| **Gene*** | **Number of detected genes** | | | | |
| --- | --- | --- | --- | --- | --- |
|  | **8005** | **CECT-4245** | **JF2635** | **TM12** | **TM18** |
| T2SS_gspD | 2 | 2 | 2 | 2 | 2 |
| T2SS_gspE | 1 | 1 | 1 | 1 | 1 |
| T2SS_gspF | 1 | 1 | 1 | 2 | 2 |
| T2SS_gspG | 1 | 1 | 2 | 1 | 1 |
| T2SS_gspM | 1 | 1 | 1 | 1 | 1 |
| T2SS_gspH | 1 | 1 | 1 | 1 | 1 |
| T2SS_gspI | 1 | 1 | 1 | 2 | 1 |
| T2SS_gspJ | 1 | 1 | 1 | 1 | 1 |
| T2SS_gspK | 1 | 1 | 1 | 1 | 1 |
| T2SS_gspL | 1 | 1 | 1 | 1 | 1 |
| T2SS_gspN | 1 | 1 | 1 | 1 | 1 |
| T2SS_gspO | 0 | 0 | 0 | 0 | 0 |
| T2SS_gspC | 1 | 1 | 1 | 1 | 1 |
| T4SS_virb4a | 0 | 0 | 1 | 0 | 0 |
| T4SS_t4cp1a | 0 | 0 | 1 | 0 | 0 |
| T4SS_MOBBa | 2 | 1 | 3 | 0 | 0 |
| T1SS_omf | 5 | 5 | 4 | 4 | 5 |
| T1SS_mfp | 2 | 2 | 2 | 1 | 1 |
| T1SS_abc | 7 | 7 | 8 | 6 | 7 |
| T5aSS_PF03797 | 1 | 1 | 1 | 0 | 0 |
| T4P_pilT_pilU | 3 | 3 | 3 | 3 | 3 |
| T4P_pilP | 1 | 1 | 1 | 1 | 1 |
| T4P_pilQ | 1 | 1 | 2 | 1 | 1 |
| T4P_pilAE | 6 | 6 | 4 | 5 | 6 |
| T4P_pilB | 2 | 2 | 2 | 2 | 2 |
| T4P_pilC | 2 | 2 | 2 | 1 | 1 |
| T4P_pilI_pilV | 1 | 1 | 1 | 1 | 1 |
| T4P_pilN | 1 | 1 | 1 | 1 | 1 |
| T4P_pilO | 1 | 1 | 1 | 1 | 1 |
| T4P_pilM | 2 | 2 | 2 | 2 | 2 |
| T4P_pilD | 1 | 1 | 1 | 1 | 1 |
| T9SS_porV | 0 | 0 | 0 | 0 | 0 |
| T9SS_sprE | 0 | 0 | 0 | 0 | 0 |
| T9SS_sprA_PF14349 | 1 | 1 | 1 | 1 | 1 |
| T9SS_gldN_TIGR03523 | 0 | 0 | 0 | 0 | 0 |
| T9SS_gldK_TIGR03525 | 0 | 0 | 0 | 0 | 0 |
| T9SS_sprT | 0 | 0 | 0 | 0 | 0 |
| T9SS_gldM_TIGR03517 | 0 | 0 | 0 | 0 | 0 |
| T9SS_gldL_TIGR03513 | 0 | 0 | 1 | 0 | 0 |
| T9SS_gldJ_TIGR03524 | 0 | 0 | 0 | 0 | 0 |
| T9SS_porU | 0 | 0 | 0 | 0 | 0 |
| T9SS_porQ | 0 | 0 | 0 | 0 | 0 |
| T3SS_sctC | 0 | 0 | 0 | 0 | 0 |
| T3SS_sctJ | 0 | 0 | 1 | 0 | 0 |
| T3SS_sctN | 2 | 2 | 3 | 2 | 2 |
| T3SS_sctS | 0 | 0 | 1 | 0 | 0 |
| T3SS_sctR | 0 | 0 | 1 | 0 | 0 |
| T3SS_sctQ | 0 | 0 | 1 | 0 | 0 |
| T3SS_sctV | 0 | 0 | 1 | 0 | 0 |
| T3SS_sctU | 0 | 0 | 1 | 0 | 0 |
| T3SS_sctT | 0 | 0 | 1 | 0 | 0 |
| T6SSi_tssA | 2 | 2 | 0 | 0 | 0 |
| T6SSi_evpJ | 4 | 4 | 0 | 1 | 0 |
| T6SSi_tssB | 1 | 1 | 0 | 0 | 0 |
| T6SSi_tssC | 1 | 1 | 0 | 0 | 0 |
| T6SSi_tssD | 4 | 4 | 0 | 0 | 0 |
| T6SSi_tssE | 1 | 1 | 0 | 0 | 0 |
| T6SSi_tssF | 1 | 1 | 0 | 0 | 0 |
| T6SSi_tssG | 1 | 1 | 0 | 0 | 0 |
| T6SSi_tssH | 2 | 2 | 1 | 1 | 1 |
| T6SSi_tssI | 4 | 4 | 0 | 0 | 0 |
| T6SSi_tssJ | 1 | 1 | 0 | 0 | 0 |
| T6SSi_tssK | 1 | 1 | 0 | 0 | 0 |
| T6SSi_tssL | 1 | 1 | 0 | 0 | 0 |
| T6SSi_tssM | 1 | 1 | 0 | 0 | 0 |
| Flg_sctJ_FLG | 1 | 1 | 2 | 1 | 1 |
| Flg_sctS_FLG | 1 | 1 | 2 | 1 | 1 |
| Flg_sctN_FLG | 2 | 2 | 3 | 2 | 2 |
| Flg_flgB | 1 | 1 | 2 | 1 | 1 |
| Flg_sctQ_FLG | 2 | 2 | 3 | 2 | 2 |
| Flg_sctT_FLG | 1 | 1 | 2 | 1 | 1 |
| Flg_sctU_FLG | 1 | 1 | 2 | 1 | 1 |
| Flg_fliE | 1 | 1 | 2 | 1 | 1 |
| Flg_sctR_FLG | 1 | 1 | 2 | 1 | 1 |
| Flg_flgC | 1 | 1 | 2 | 1 | 1 |
| Flg_sctV_FLG | 1 | 1 | 2 | 1 | 1 |
| Tad_rcpA | 0 | 0 | 0 | 0 | 0 |
| Tad_tadZ | 2 | 2 | 2 | 2 | 2 |
| Tad_tadV | 0 | 0 | 0 | 0 | 0 |
| Tad_tadC | 0 | 0 | 0 | 0 | 0 |
| Tad_tadB | 0 | 0 | 0 | 0 | 0 |
| Tad_tadA | 0 | 0 | 0 | 0 | 0 |
| Tad_tadF | 0 | 0 | 0 | 0 | 0 |
| Tad_tadE | 0 | 0 | 0 | 0 | 0 |
| Tad_flp | 0 | 0 | 0 | 0 | 0 |
| T4SS_virb4b | 0 | 0 | 0 | 0 | 0 |
| T4SS_t4cp1b | 0 | 0 | 0 | 0 | 0 |
| T4SS_MOBBb | 0 | 0 | 0 | 0 | 0 |
| T4SS_F_traB | 0 | 0 | 1 | 0 | 0 |
| T4SS_F_traF | 0 | 0 | 1 | 0 | 0 |
| T4SS_F_traG | 0 | 0 | 1 | 0 | 0 |
| T4SS_F_traE | 0 | 0 | 1 | 0 | 0 |
| T4SS_F_trbC | 0 | 0 | 1 | 0 | 0 |
| T4SS_F_traK | 0 | 0 | 1 | 0 | 0 |
| T4SS_F_traH | 0 | 0 | 1 | 0 | 0 |
| T4SS_F_traN | 0 | 0 | 1 | 0 | 0 |
| T4SS_F_traL | 0 | 0 | 1 | 0 | 0 |
| T4SS_F_traV | 0 | 0 | 1 | 0 | 0 |
| T4SS_F_traW | 0 | 0 | 1 | 0 | 0 |
| T4SS_T_virB1a | 0 | 0 | 0 | 0 | 0 |
| T4SS_F_traU | 0 | 0 | 1 | 0 | 0 |
| T4SS_G_tfc7 | 0 | 0 | 0 | 0 | 0 |
| T4SS_virb4 | 0 | 0 | 0 | 0 | 0 |
| T4SS_t4cp1 | 0 | 0 | 0 | 0 | 0 |
| T4SS_MOBB | 0 | 0 | 0 | 0 | 0 |
| T4SS_T_virB11 | 0 | 0 | 0 | 0 | 0 |
| T4SS_T_virB6 | 0 | 0 | 0 | 0 | 0 |
| T4SS_T_virB5 | 0 | 0 | 0 | 0 | 0 |
| T4SS_T_virB10 | 0 | 0 | 0 | 0 | 0 |
| T4SS_T_virB3 | 0 | 0 | 0 | 0 | 0 |
| T4SS_T_virB2 | 0 | 0 | 0 | 0 | 0 |
| T4SS_T_virB1 | 0 | 0 | 1 | 0 | 0 |
| T4SS_T_virB9 | 0 | 0 | 0 | 0 | 0 |
| T4SS_T_virB8 | 0 | 0 | 0 | 0 | 0 |

*T[x]SS: Type [x] protein secretion system. T4P : Type IV pilus. Flg: Flagellum. Tad: Tight adherence secretion system.

Supplementary Figures


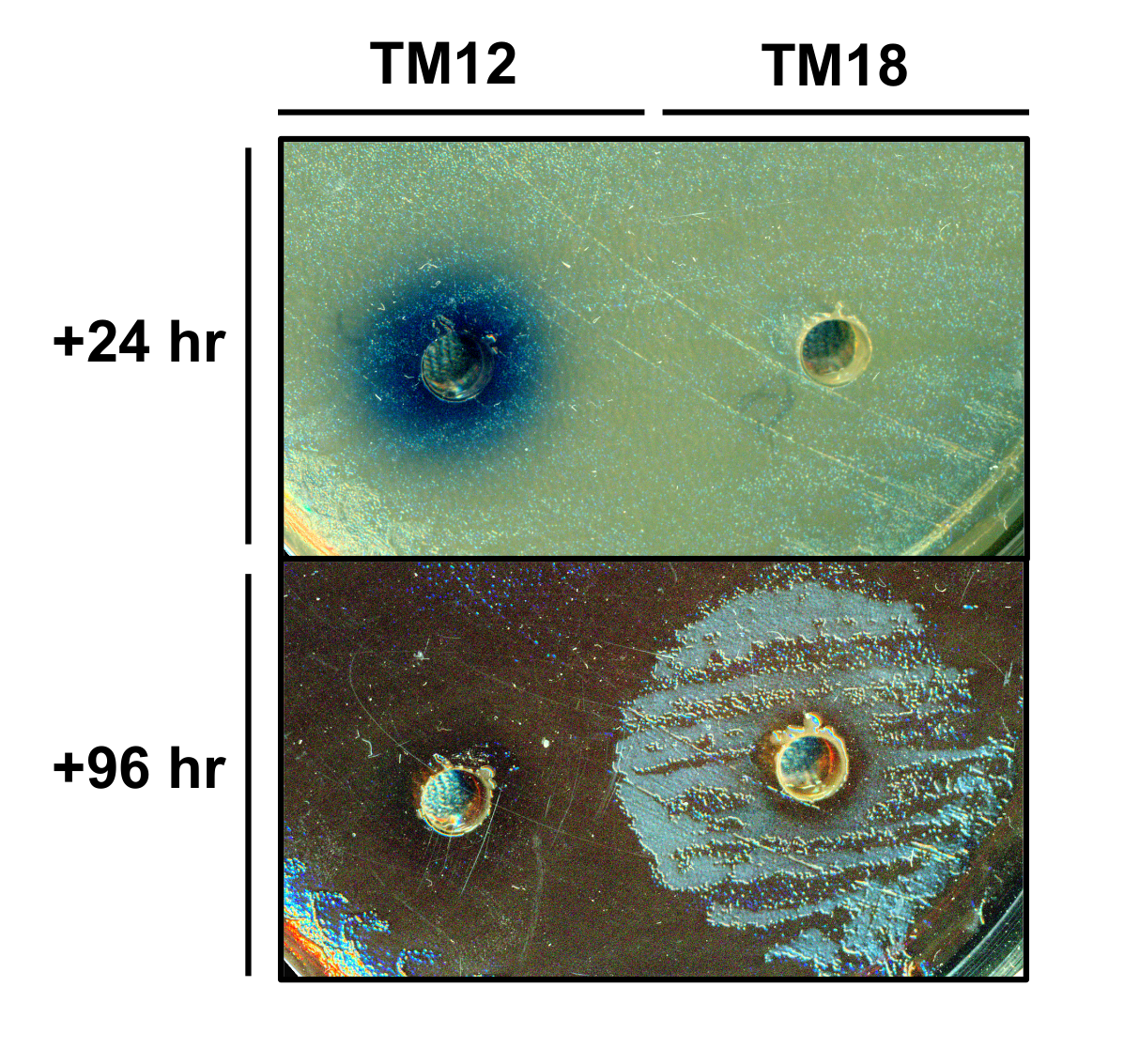


## Supplementary Figure 1. Diffusible inhibitory effect of *A. sobria* strains TM12 and TM18 against *A. salmonicida* subsp. *salmonicida* 09-0167 lawns on TSA plates. Left : Production of a blue pigment by TM12 (24 hr) which overlaps a radial inhibition area on *A. salmonicida* subsp. *salmonicida* lawns (96 hr). Right: a fairly larger inhibition area is produced by TM18 (96 hr), even without production of the blue pigment as seen in TM12 (24 hr).


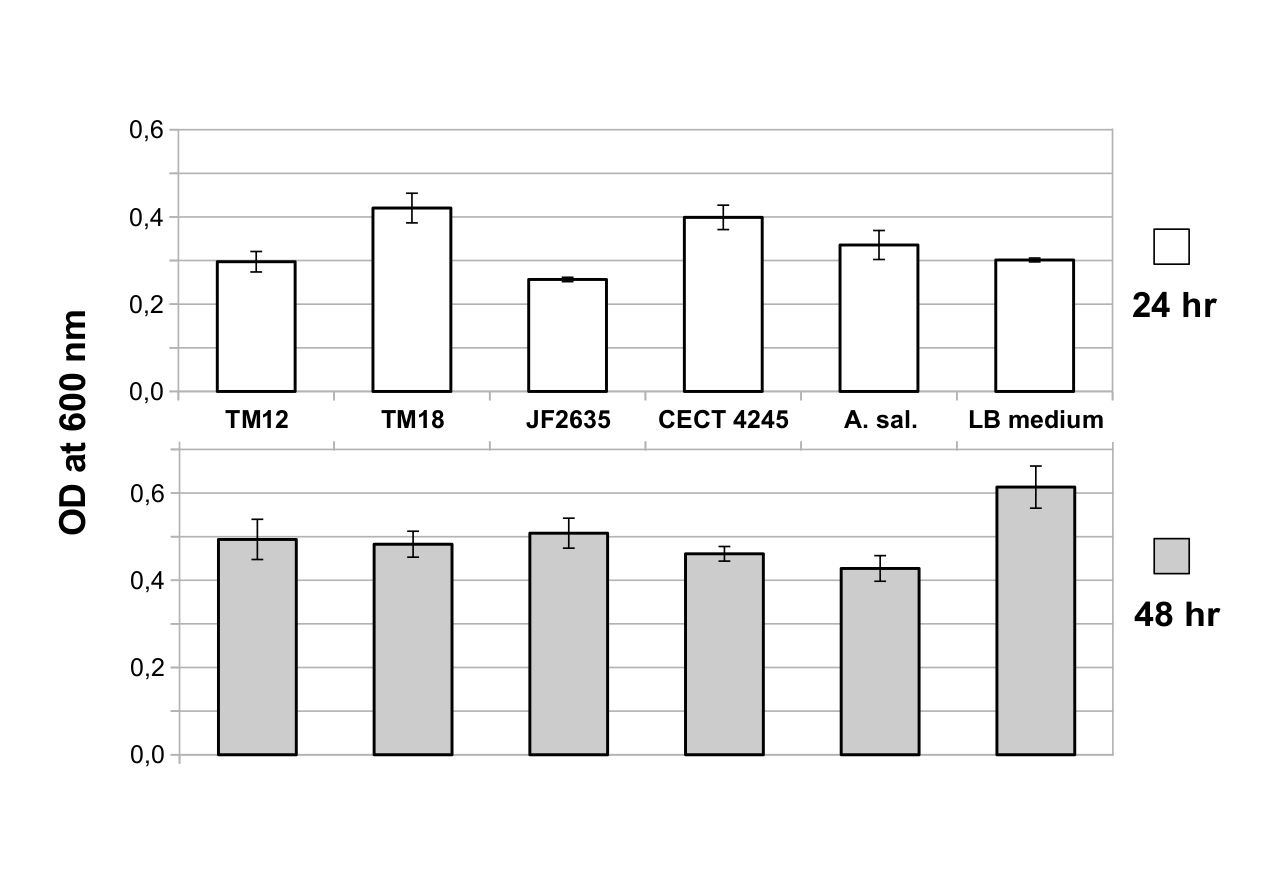


## Supplementary Figure 2. Growth of *A. salmonicida* 01-B526 in [3:1] mixtures of LB broth and *A. sobria* culture supernatants (CS). Solid bars and vertical segments indicate the mean OD_600_ and standard error, respectively. A. sal., *A. salmonicida* 01-B526 CS (neutral control). This experiment was conducted in triplicates.

##
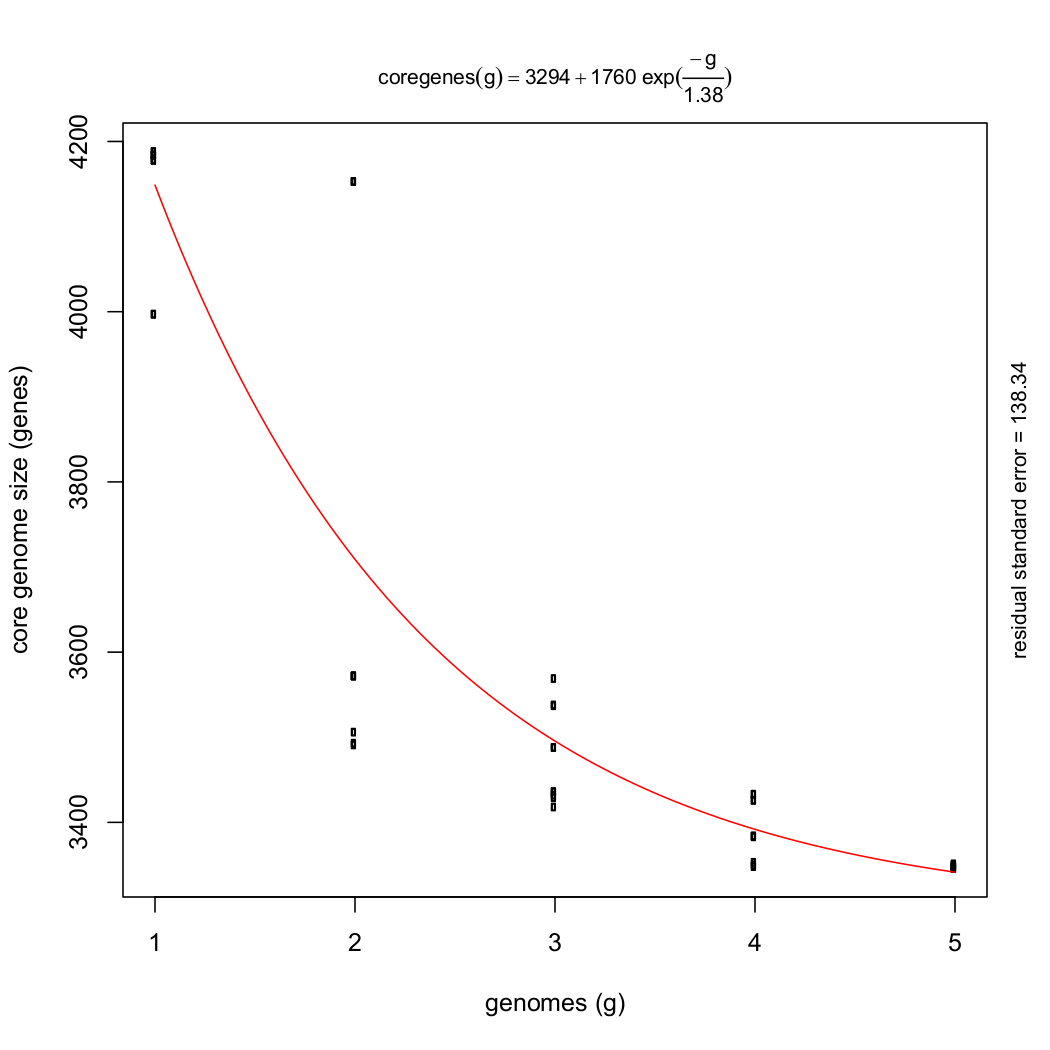


Supplementary Figure 3A. Development plot of the *A. sobria* core genome. Points indicates the number of core genes (*i.e.* common homologous gene clusters) for all possible subsets of {1, 2, 3, …, n} genomes among all five included in our study. Red line indicates the regression curve (equation shown above the plot). Residual standard error (shown at the right of the plot) is expressed in number of genes.


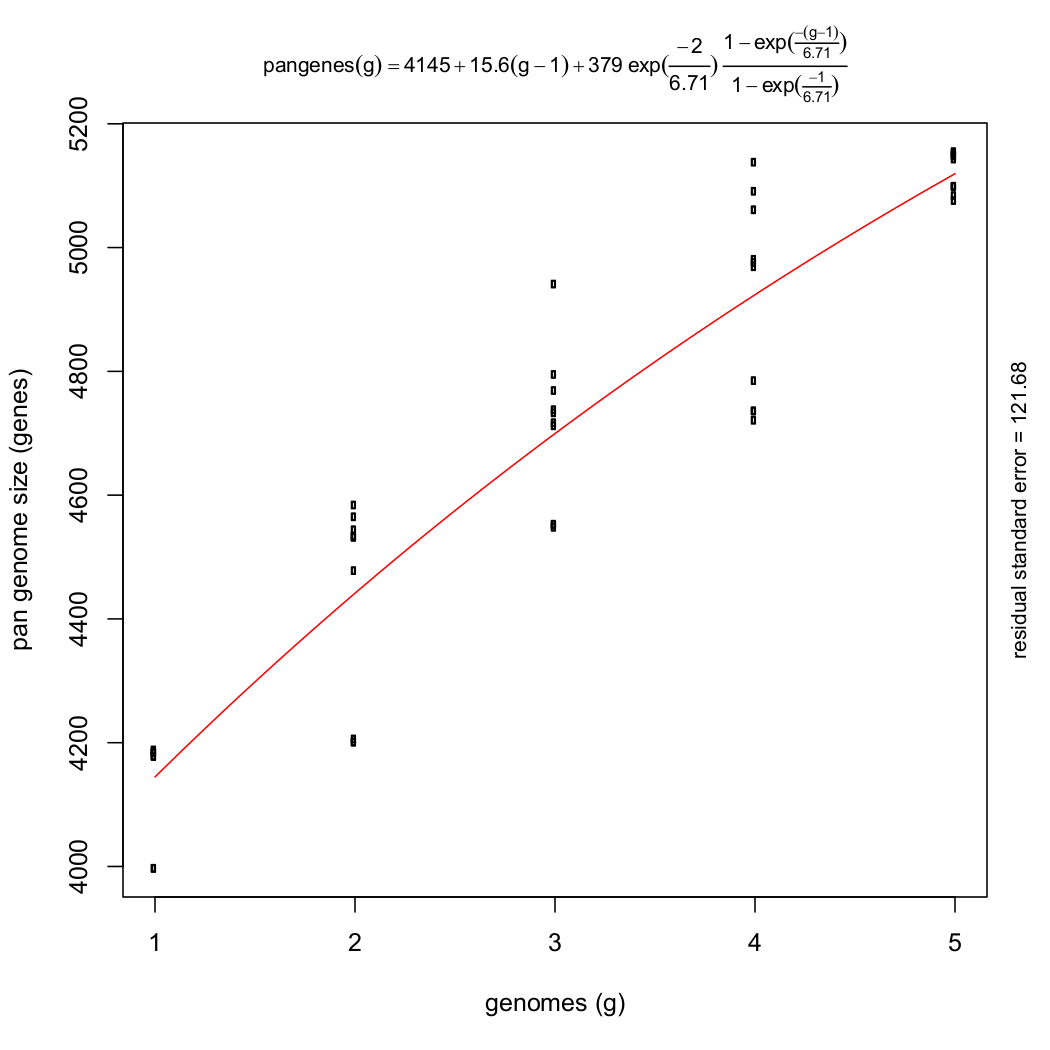


Supplementary Figure 3B. Development plot of the *A. sobria* pangenome. Points indicates the number of pangenes (*i.e.* total nonredundant homologous gene clusters) for all possible subsets of {1, 2, 3, …, n} genomes among all five included in our study. Red line indicates the regression curve (equation shown above the plot). Residual standard error (shown at the right of the plot) is expressed in number of genes.


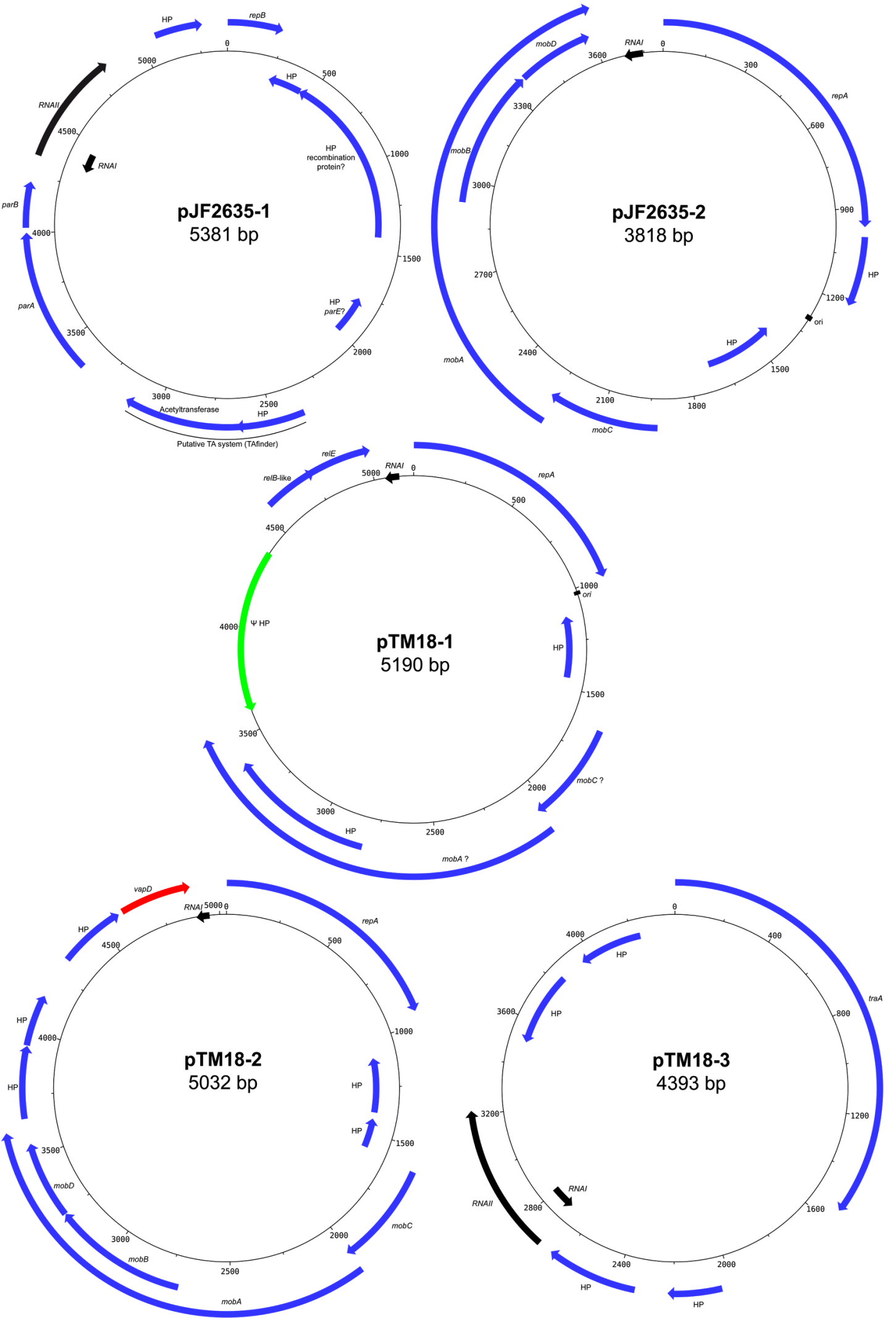


## Supplementary Figure 4. Plasmidome of the *A. sobria* isolates. HP: hypothetical protein. Blue arrows: coding DNA sequences (CDS) that encode proteins involved in plasmid mobility or hypothetical proteins. Red arrows: putative virulence factors. Green arrow: pseudogene. Black arrows: genes encoding small regulatory RNAs involved in ColE1- and ColE2-type replication.


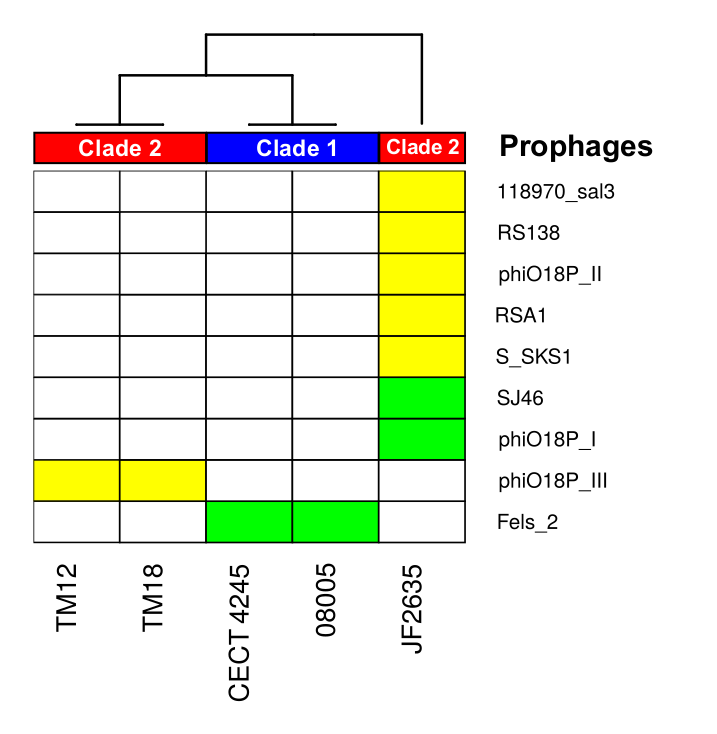


## Supplementary Figure 5. Heatmap of predicted phage elements in *A. sobria* genomes. Yellow: presumably incomplete prophages. Green: presumably intact prophages. White: prophage is absent. “Clade 1” and “Clade 2” refers to clades established in the molecular phylogeny of the *Aeromonas* core genome and average nucleotide identity (ANI) analysis.
